# Local-province chief executive officer and managerial myopia: Evidence from China

**DOI:** 10.3389/fpsyg.2022.966996

**Published:** 2022-09-13

**Authors:** Qian Chen, Xiang Gao, Shuzhen Niu, Xiao Wang, Qian Wei

**Affiliations:** ^1^Department of Economics, Shenzhen MSU-BIT University, Shenzhen, China; ^2^Research Center of Finance, Shanghai Business School, Shanghai, China; ^3^School of Business, Sanda University, Shanghai, China; ^4^Peking University HSBC Business School, Shenzhen, China; ^5^School of International Economics and Trade, Guangxi University of Foreign Languages, Nanning, China

**Keywords:** manager myopic behavior, local-province CEO, provincial social capital, government innovation subsidy, corporate governance

## Abstract

Managerial myopia occurs when executives value short-term benefits to the extent that firm long-run development will be obstructed. Recent studies have shown that the locality effect plays an important role in managerial myopia—local United States chief executive officers (CEOs) who work near their home states are less likely to behave myopically because of more effective monitoring and greater reputation concern. In an emerging market, government policies play a more important role in the strategic planning enterprises. A local CEO may have better understanding of local government’s policies thus makes less short-term decisions. This article adds to this literature by testing whether local-province CEOs in China, i.e., the CEO’s native place or birthplace is in the same province as her company’s headquarters, are also far-sighted. Using data on 470 publicly listed non-state-owned Chinese firms from 2014 to 2018, supportive evidence has been found that non-local-province CEOs in China tend to cut R&D expenses for beating analyst forecasts, reversing earnings decline, or pursuing higher returns. This article also confirms social capital as one mechanism of Chinese local-province CEOs behaving less myopically. This investigation also adds to the literature by revealing a new mechanism that CEO locality in China has a positive and direct bearing on how governments support corporate innovation.

## Introduction

It is a challenging task for most companies to balance long-term and short-term benefits. Graham et al. (2005) interview several hundreds of American chief executive officers (CEOs) and find that 55% of CEOs will give up a long-term project with a positive net present value (NPV) if the investment hinders them for achieving their short-term goals. Managers may emphasize short-term benefits instead of long-term benefits to seek self-interests or meet shareholders’ particular needs.

Given this scenario, managerial myopia occurs. An important manifestation of managerial myopia in Chinese non-financial firms is that they become unwilling to innovate. There exists a tendency for them to invest in the financial and real property sectors for short-term gains, rather than in long-run research and development (R&D) activities. Sen and Dasgupta (2015) point out that the management’s excessive pursuit of short-term profit maximization determines the firm’s investment decision, thus resulting in larger holdings of financial assets by companies in a developing country like India. Guo and Qing (2018) analyze the heterogeneity of financialization of China’s non-financial firms and find that after the 2008 Global Financial Crisis these firms have grown to a level equivalent size to or even higher than peers from developed countries including the United Kingdom, United States, and Japan. Specifically, China’s non-financial firms have higher interest income and expenditure due to increased borrowing and lending. This implies that managerial myopia is a prevalent phenomenon among Chinese non-financial firms.

Managerial myopia obstructs the long-term development of companies, and the “quick money-making” business model increases the insolvency risk faced by companies. Therefore, prior studies attempt to find practical solutions to prevent myopic decisions of the management. Bertrand and Schoar (2003) find that managers’ characteristics help to form their management style, which in turn has an impact on the operation and performance of firms. For example, when the Chinese CEO is older or a female, the leverage ratio of a company will be lower (Chen et al., 2014). The same dialect spoken by both the chairperson and CEO in a Chinese company can reduce agency costs significantly, and this reduction becomes more prominent if the dialect is only used by the minority (Dai et al., 2016). Lai et al. (2020) is the first to identify that non-local CEOs are more likely to reduce R&D expenses for short-term goals than the corresponding hometown CEOs. They also discover the difference to be more significant in regions and states known for higher social capital.

In the last few years, according to the Scopus database, there emerge more than 100 articles that focus on the causes and consequences of myopia for managers in different sectors. Therefore, this article investigates whether local-province CEOs are less subject to myopic behaviors than the non-locals in China, which is referred to as locality effect. There are two reasons why investigating this phenomenon in China is the interest of this article. First, it is noticed that the Chinese companies hire a higher proportion of local CEOs than the United States companies, that is more than 43.2% in the sample in this article compared to 21.3% in Lai et al. (2020)’s sample. Will the locality effect on managerial myopia be strengthened with more common local manager in a society like China, which attaches great importance to social connection? Second, as China’s economy enters the ‘new normal’ stage, Chinese government values innovation more than ever. The total social investment in R&D increased from around 1.8% of GDP in 2010 to 2.4% in 2020. It is interesting to look at the effect of public policy on innovation from a micro perspective.

The records of Chinese publicly listed private-owned companies over the period of 2014–2018 is examined and it is tested that whether, how, and why local-province CEOs behave less myopically than non-local-province CEOs. The finding is that, compared to local-province CEOs, non-local-province CEOs incline to reduce R&D expenses for beating analyst consensus, reversing earnings decline, or pursuing higher earnings. Companies hiring locals as CEOs are associated with lengthened investment horizons. The CEO locality effect is strengthened in regions with larger social capital but is suppressed by greater extent of government support for corporate innovation. The causal relationship between local-province CEO and managerial myopia is identified by resorting to the difference-in-differences (DID) method and by analyzing the consequences of a CEO turnover from locals to non-locals. Moreover, the conclusions remain unchanged after a list of robustness checks such as adopting alternative independent variable, excluding data from certain unique Chinese provinces, etc. Specifically, the local-province effect is examined instead of a finer granularity due to two reasons. First, it is common in China that the mobility of high-level human resources is high, especially within a province. Due to the imbalance allocation of education resources within a province and the barrier of College Entrance Examination, people are more likely to relocate to a different city within their home province. The major cities in home province are also people’s first choice when planning for their career development. Second, it is difficult to identify CEOs’ home cities. This difficulty shrinks our sample size to around one tenth of the size of sample using the provincial level data.

Besides providing evidence for the China case, this article extends Lai et al. (2020) by introducing a novel mediating variable to capture the extent of government support for corporate innovation. It is conjectured that the CEO locality effect will be weakened in provinces where the local government subsidize corporate innovation a lot. Therefore, the result also supplements the discussion about the impact of government’s subsidies on enterprise’ innovation. In general, the results of this article help to understand the complementary relationship between formal and informal institutions. Formal institutions such as equity ownership, employment agreement, and severance agreement can mitigate short-termism, but the associated financial costs are also quite high if companies grant stocks to CEOs or sign employment and severance contracts with them. Hiring locals as CEOs is a typical informal approach that can also curb short-termism by avoiding additional costs and at the same time saving social resources. It is shown that CEOs’ native place or birthplace is a crucial personal trait, which plays a role in the process of CEO management style formation, thus providing additional empirical evidence to support the argument by Bertrand and Schoar (2003).

This article contributes to the body of research that explores factors affecting managerial myopia. The potential determinants are divided into three categories. The first category includes a collection of market factors, such as capital market, accounting system, hostile takeover, and analysts. Stein (1988, 1989) proposes that the capital market is short-term oriented and stimulates managers to behave myopically. However, Jensen (1988) believes that there is no evidence that the capital market is short-term oriented, and he claims that the capital market is active and punishes managerial myopia. Zhong et al. (2017) argue that accounting conservatism exerts an impact on managerial myopia, and eventually can affect the innovation ability of companies. Companies evaluate projects before making investment decisions, and in general long-term investments are risky and costly. On the one side, accounting conservatism raises managers’ awareness of risks, so managers become more cautious when making investment decisions, promoting managerial short-termism. On the other side, accounting conservatism increases the proportion of R&D expenditures to be expensed, and lowers short-term earnings as well as managers’ salaries, stimulating managerial myopia. Also, Brochet et al. (2015) find that reducing the frequency of financial reporting can also effectively curb managerial short-termism. Stein (1988) thinks that the pressure from hostile takeover forces managers to accomplish short-term goals at the cost of the long-term benefits of companies. That is because companies’ dismal performance incurs of undervaluation of share price, increasing the probability of being acquired at a low price. However, some scholars believe that hostile takeovers threat can effectively prevent managers from being lazy, promoting corporate innovation. Atanassov (2013) finds that the number of patents applied or cited by listed companies decreases in the states that passed the anti-takeover act. Some scholars believe that analysts put too much pressure on managers. To meet market expectations, managers sometimes have to give up a long-term project with a positive NPV if the investment leaves the company unable to achieve its short-term goals. He and Tian (2013) confirm that a higher analyst coverage of the listed company may lead to a smaller number of patent applications and patent citations.

The second category is called corporate factors, such as ownership concentration, institutional ownership, executive ownership, employment and severance contract, compensation contract, independent directors, leverage, and Return on Equity (ROE). The interests of shareholders and managers are not consistent, and the decentralization of equity may cause managers to become more short-term oriented. Bange and De Bondt (1998) point out that if shares are dispersed, shareholders may lose control over the company. Thus, they may not have the ability to restrain the behaviors of managers. Bushee (1998) finds that managers are less likely to reduce R&D expenses for reversing earnings decline if institutional investors hold a large proportion of shares of listed companies, as institutional investors are experienced and play supervisory roles in the capital market. However, it is believed that the high turnover rate and the momentum strategy of institutional investors make them value more on short-term benefits, inducing a shorter investment horizon of related companies. Yan and Zhang (2009) classify long-term and short-term institutional investors according to their past 4-quarter portfolio turnover rate. Tucker (2018) validates the use of turnover rate as a measure of fund short-termism. Granting shares to executives can align the benefits of agents and principals and prompt agents to concentrate on the long-term development of companies. It is an effective solution to mitigate managerial myopia. Gonzalez-Uribe and Groen-Xu (2017) provide evidence for the impact of employment agreements on corporate innovation—for one additional year of CEOs’ tenure, the number of patents cited by companies increases by 8%. Long-term employment contracts between companies and CEOs facilitate corporate innovation by increasing R&D expenditures, hiring more researchers, and providing them excellent packages. The above conclusion can be verified by the case of Japanese companies. Most Japanese companies implement career-long employment, and this mechanism enhances employees’ feelings of belonging. The interests of employees are closely connected with the long-term benefits of companies, which is conducive to the sustainable development of companies. Career-long employment has much helped Japanese economic recovery after World War II. Holmstrom (1982) points out that complete compensation contracts can align the interests of managers and shareholders, inhibiting managerial myopia. Thus, an incomplete compensation contract is a cause of managerial myopia. Bolton et al. (2006) believe that the compensation contract emphasizing the short-term performance of companies induces managers to behave myopically and increase the speculative component of stock prices. Wu and Wu (2010) find that the compensation contract of listed companies in China cannot solve the principal-agent problem and self-interested managers increase agency cost, which eliminates the incentive effect of compensation contracts. Osma (2008) confirms that the independent director can effectively restrain the actions of companies and curb short-termism if she has enough professional knowledge to identify the speculative behaviors of companies. If the company can raise capital by debt financing, the solvency and profitability of the company are relatively good (Valaskova et al., 2021b). Besides, creditors supervise the investment decisions of the company after financing, reducing flexibility for earning management. Brochet et al. (2015) believe that companies with low profitability engage in myopic behaviors to explain relatively poor performance to outsiders. Lastly, Xu and Liu (2020) emphasize the impact of intellectual capital on firm performance.

The third category consists of individual manager characteristics including experience, age, tenure, and native place or birthplace. In terms of experience, Narayanan (1985) finds that the more experienced the manager is, the more her ability is known to outsiders, which weakens motivations for managerial myopic caused by information asymmetry. As for age, the older the manager is, the more defensive and conservative she becomes when making investment decisions, which stimulates managerial myopic. Gibbons and Murphy (1992) argue that career concern is a significant implicit incentive, and managers pay attention to the impact on future earnings when making investment decisions. Younger and overconfident managers prefer long-term projects, while managers who are close to retirement and have less confidence may be more short-term oriented (Bukalska, 2020; Liang et al., 2020). Lundstrum (2002) believes that the R&D expenditures of companies decrease with CEOs’ age. Wu and Li (2012) also prove that CEOs’ age is positively correlated with managerial myopia. Tenure is also an important aspect. Dechow and Sloan (1991) find that companies invest significantly less in R&D activities in the CEOs’ last year of office. Rao et al. (2012) propose that there is an inverted U-shape relation between CEOs’ phase of tenure and investment horizon. Specifically, CEOs in the early and late terms are likely to act myopically, while CEOs in the mid-career have long investment horizon. This article follows most closely the literature highlighting the CEO birthplace. Lai et al. (2020) identify that CEOs’ native place has an influence on managerial myopia, and local-province CEOs are less likely to be short-term oriented based on a United States sample. This effect becomes more evident when the area that the company locates in has more social capital or better governance quality (Khyareh and Amini, 2021). All in all, our article explores an important issue of CEO locality, which provides a perspective that could link the above-mentioned external and internal categories of determinants of managerial myopia behaviors.

Moreover, this article also has implications for measuring managerial myopia. The definition of managerial myopia reveals that when managers confront a tradeoff between short-term goals and long-term investments, they place more weight on short-term goals. Therefore, it is necessary to find a proxy for long-term investment before measuring managerial myopia. R&D expenditure is commonly used as a proxy for long-term investments since there is a time lag between investments in R&D activities and future earnings they will bring (Dobrzanski et al., 2021). The Chinese Accounting Standards stipulate that a few R&D expenditures in the development phase of internal R&D projects can be capitalized when certain conditions are met. However, capitalization conditions are rigorous. Some scholars use capital expenditure as a proxy for long-term investments, but this measurement is not as proper as R&D expenditures. Firstly, compared with R&D expenditure, capital expenditure has a smaller impact on the current earnings. This is because capital expenditures are first capitalized when they are invested, and depreciation or amortization expenses are accrued annually during the subsequent accounting periods. Secondly, current cash flows are influenced more by R&D expenditure than by capital expenditure. Himmelberg and Petersen (1994) put forward that companies commonly use their capital to fund R&D activities since R&D projects lack collateral assets and suffer from asymmetric information, while capital expenditures can be financed by external funds, such as bank loans and pledged bonds. Thirdly, capital expenditures maintain a high degree of liquidity and can be sold for cash flows. However, investments for R&D activities will become sunk costs if they fail to generate revenue in the future.

When it comes to connecting R&D activities with myopia measurement, some studies use R&D intensity (i.e., the ratio of R&D expenses to revenues or total assets). The higher the R&D intensity is, the longer the manager’s investment horizon is, thus, there are fewer myopic behaviors. However, this measurement is not accurate to capture the trade-off in the myopic behavior of managers. Managerial myopia is one of the behaviors of earnings management, and managers accomplish short-term goals (pursuing higher performance, avoiding earnings decline, reducing the scale of losses or beating analysts’ consensus forecasts) at the cost of long-term benefits (reducing R&D expenses). Bushee (1998) uses earnings before tax and R&D as short-term performance indicators. By comparing this indicator between two consecutive years, the companies are divided into three categories to explore whether the relationship between institutional investors’ shareholding ratio and probabilities of CEOs cutting R&D expenditures changes with different short-term goals. The underlying assumption is that CEOs can estimate without bias this year’s earnings before tax and R&D as early as possible and make corresponding decisions about whether to reduce R&D expenditures.

The rest of this article is organized as follows. The Section “Literature review and hypothesis” develops the hypothesis based on the existing relevant literature. The Section “Empirical methodology” first introduces data sources, sampling methods, and empirical specifications, and then discusses the construction of main variables. In the Section “Results,” the baseline empirical results are presented, followed by a variety of robustness tests. Finally, the Section “Conclusion” concludes and points out both the theoretical and practical significance of this article.

## Literature review and hypothesis

This article attempts to investigate the CEO locality effect and focuses on the self-interest motivation for managerial myopia in Chinese privately owned firms.

To begin with, the principal-agent theory lays down the foundation for developing our hypotheses. Jensen and Meckling (1976) propose the principal-agent theory. If both principals and agents maximize their utility, then agents do not always take actions as principals’ wishes. The agency problem is derived from the modern company system separating ownership from operations. On the one hand, shareholders and managers have different utility functions. Owners of the company expect that managers work with due diligence and maximize shareholders’ value, while managers want to maximize their utilities such as higher salary, more paid vacation, higher social reputation, and lower occupational risk. On the other hand, shareholders cannot know every aspect of operations of the company, which enables managers to make use of information superiority to maximize their utilities like increasing their salary by earnings management. Jensen and Meckling (1976) believe that principals can limit the behaviors of agents by establishing incentive and supervision institutions so that agents can take actions as principals’ wishes. This article distinguishes between local versus non-local mangers as the agent of local companies and stakeholders as the principal.

Managers may exhibit short-sightedness due to three reasons. Firstly, the wage distortion hypothesis indicates that the asymmetric information in the labor market leads to managerial myopia. Most directors of listed companies come from the region where the company is located, and they have a better knowledge of the ability and character of CEOs who were born or grew up in company locations. They either have established direct contact with local-province CEOs or have indirectly learned the local-province CEOs’ information through social networks. However, the board of directors knows much less about the ability and the character of non-local-province CEOs, and they update the belief of non-local-province CEOs’ ability and determine non-local-province CEOs’ future income according to the current performance. Hence, non-local-province CEOs have the opportunity to pretend to be high-able persons by acting myopically and gain high salaries.

Secondly, the high mobility of non-local-province CEOs forced them to be more short-term oriented. Non-local CEOs seize on excellent short-term performance as proof of their high abilities and move to a new company before the impact of the myopic decision is revealed. However, the horizons of local-province CEOs are longer since they are attached to hometowns and dislike relocation. Hu (2018) finds that companies hiring locals as CEOs release less voluntary disclosure and conceal more bad news, which is consistent with the concern that local-province CEOs dislike relocating.

Thirdly, we turn to the place attachment theory, which is developed by interdisciplinary research of geography and psychology. Tuan (1975) observes that there seems to be a special attachment relationship between people and some places. The definition of place attachment theory reveals “the emotional connection produced by the interaction between people and places.” The most classical version of place attachment theory is a two-dimension hypothesis, including place dependence and place identity. Place dependence emphasizes individuals’ dependence on locations as places provide resources and facilities for the activities of individuals, while place identity emphasizes individuals’ mental attachment to the local spirit. Bricker and Kerstetter (2000) add on it with the third dimension—the lifestyle, which refers to the interaction between individuals and locations in daily life, resulting in deep emotional connections. This theory can be extended to contain more components, such as those based on the degree of bonding character and the degree of bonding intensity, covering place familiarity, place belongingness, place identity, place dependence and place rootedness. At present, place attachment theory is widely used in the field of natural resources management, such as national parks and natural heritage sites. Halpenny (2010) explores the relationship between place attachment and environmental protection and treats 355 tourists in the Canadian National Park as subjects. The results show that place attachment contributes to environmental behaviors. In this article, place attachment theory is applied to the field of economics. In specific, people’s attachment to their hometowns can generate reputation pressure, and hence can affect myopic behaviors. Place attachment theory suggests that there are emotional connections between people and their native places or birthplaces, and people are concerned about the welfare of the people in their hometown (Fullilove, 1996). Most people view their hometown as a holy place and like maintaining a good reputation in their hometown (Relph, 1976). Therefore, the utility function of local-province CEOs contains reputation, and it accounts for a high proportion in the utility function. If local-province CEOs behave myopically, the compensation they get from the improved short-term performance will not outweigh the loss of reputation, eventually resulting in reduced net utilities. Therefore, Stein (1989) thinks that local CEOs faced less market pressure related to short-term results. Based on the above analysis, the underlying assumption is proposed:

*H1: Local-province CEOs are less myopic than non-local-province CEOs in China.*

Next, this article explores the mediating factors for the CEO locality effect. One of the transmission mechanisms is that people’s attachment to their hometown can generate reputation pressure, and hence can affect myopic behaviors. However, the strength of attachment varies – some people might be more connected to their hometowns, while others are less so. Lai et al. (2020) argue that people are more closely connected in regions with high social capital. If the province where the company headquarters is located has more social capital, local-province CEOs are more closely associated with the residents of the province and accordingly face higher reputation pressure. Thus, their horizon is further longer. Therefore, the following hypothesis is proposed.

*H2: The CEO locality effect is stronger if the province where the company headquarters is located has more social capital than other provinces.*

The CEO locality effect functions through the decision-making process (informal institutions) rather than rules and regulations (formal institutions). It is believed that informal institutions and formal institutions are complementary. If formal institutions are perfect, the role of informal institutions will be less critical. There also exists evidence that if the marketization of the province where the company is located is higher or its institutional environment is better, that is, the formal institutions allocate resources more openly and transparently, the role of social networks in accessing resources will be limited, and the impact of dialect differences on gaining government subsidies will also be weakened.

According to the Chinese Entrepreneur Survey System (CESS) in 2014, 41.3% of interviewed entrepreneurs think that China lacks a social environment encouraging corporate innovation. If external formal institutions, such as the government financial supports to the R&D activities of companies, are improved, then the impact of R&D activities on short-term performance may be weakened. In this scenario, non-local-province CEOs’ tendency to cut R&D expenditure is lower, and the behaviors of local-province CEOs and non-local-province CEOs become similar, damping the CEO locality effect. The third hypothesis is hence:

*H3: The CEO locality effect is weaker if external formal institutions that can mitigate managerial myopia are improved (the company receives more government financial support for innovation)*.

Hypotheses 2 and 3 relate closely to the strand of theoretical literature on the origin of managerial myopia. The definition of managerial myopia covers three aspects: (I) the long-term projects of companies are underinvestment; (II) the underinvestment is caused by overemphasizing the short-term goals of companies; (III) the underinvestment must be a suboptimal solution, and it hurts the long-term development of companies (Cheng et al., 2005). The extant literature divides short-termism into two categories based on differing motivations. One motivation is to meet the self-interests of managers. The wage distortion hypothesis indicates that the asymmetric information in professional manager markets will lead to managerial myopia. If markets or boards of directors have limited knowledge about CEOs’ abilities, they will update the belief of CEOs’ ability and determine CEOs’ future income according to the current performance. So, the manager is more likely to sacrifice long-term benefits to boost short-term performance when she has private information (Narayanan, 1985). The takeover hypothesis and managerial entrenchment hypothesis indicate that managers have motivations to remain in office or avoid being fired after companies are acquired, and they will take defensive actions in response to threats. For example, managers can improve the short-term performance of companies by myopic strategies to lower the risk of a takeover and the occupational hazard. Another motivation is to meet the self-interests of shareholders. When a manager puts money into long-term projects, she can demand a raise in salary by threatening to leave the company before the cash flows from the projects can be recovered. To reduce this kind of hold-up losses, shareholders prefer short-term projects, promoting managers to reduce long-term investments. In practice, shareholders can control the investment behaviors of companies by signing compensation contracts that are based on short-term performance with managers (Lundstrum, 2002). Besides, ownership structure theory implies that investment horizons vary from investor to investor. Increasing the shareholding ratio of strategic investors can effectively inhibit managerial myopia (Bushee, 1998). On the contrary, if the shareholding ratio of speculative investors increases, managers may tend to invest in short-term projects to meet the interests of speculative investors.

## Empirical methodology

### Data sources and sample

This article focuses on the Chinese stock market A-share companies that are owned by private investors, as the operation and management of state-owned enterprises (SOE) are subject to administrative orders rather than monetary incentives. Zhang (2002) points out that maximizing shareholders’ value or firm value is not the primary goal for the management of SOEs. Yan and Deng (2018) show that the regulated remuneration of the CEOs of SOEs cannot significantly reduce innovation activities, meaning that SOE managers have no incentive to behave myopically for bonuses. As a result, it is unlikely to capture managerial myopia in a sample of SOEs. Besides, the appointment of SOE executives is influenced by political considerations, jeopardizing the randomness of the sample if SOEs are incorporated.

The financial data of publicly listed private-sector firms is obtained from the CSMAR and Wind database. The variable of particular interest is R&D expenditure and is used as a proxy for the emphasis put on long-term investment by executives. Since data on R&D expenditures of A-share firms before 2013 is not available, a sample period from 2014 to 2018 has been chosen.

While the whereabouts of firm headquarters are directly available in the CSMAR database, the native place or birthplace of a CEO is collected manually via three complementary methods. First, CSMAR has already disclosed the birthplace information for many CEOs. Second, the Chinese search engine Baidu.com is used to search for keywords “executive name” plus “native place” or “birthplace” or “chamber of commerce”, trying to match the information on the native places of CEOs. Third, CEOs’ ID card numbers are collected from the prospectus or other legal documents released by the company, and the first six digits of the ID card number contains information about individual’s birthplace.

As for the mediating variables, data on provincial social capital are sourced from the Report on Business Environment Index of Chinese Provinces (Wang et al., 2013, 2017); and records on the Chinese government’s financial support for corporate innovation are extracted from the annual financial reports of publicly listed firms. Specifically, firms disclose details of received government subsidy, including amounts and uses of funds, in their reports. Thus, data on government subsidies for firm R&D activities is compiled.

As for the last step, the dataset is screened according to the following conditions. First, Special Treatment (ST) and *ST companies due to irregularities in their financial positions are excluded. Then, financial institutions are deleted because their financial statements are prepared differently than firms in other industries. Next, companies that initially went public in 2017 and 2018 are also eliminated. The reason is that computing the annual change in R&D expenditures requires a minimum of two consecutive years of data. The fourth step is to exclude sample companies with missing data on R&D expenditures, CEO birthplace, financial conditions, or corporate governance indicators. Finally, 2,083 observations representing 470 unique companies are obtained, which is commonly regarded as a sufficient sample size in the literature for carrying out panel regression with approximately 10 independent variables in the model. Noticeably, the sample is an unbalanced panel as some measurements, such as financial data or CEO information may be randomly missing for some companies in some years. In fact, the unbalanced panels are the norm when dealing with data from firms or individuals, as there might be change in board or transition in CEO terms, which sometimes is not revealed in company’s annual report or any public channel. Nevertheless, robust regression estimates can still be obtained for the unbalanced data structure as long as the missing measurements are random. It merits a note that, to minimize the impact of outliers on the regression estimates, all continuous variables are winsorized at the 1% and 99% level.

### Specification and variable construction

To measure managerial myopia, first earnings before tax and R&D as well as Earnings per Share (EPS) are chosen as indicators for short-term performance, based on the discussion in Section “Literature review and hypothesis.” Managers manipulate these two performance indicators to meet the performance goals such as last year’s earnings before tax and R&D and analyst forecasts of EPS, respectively. Then, based on the earnings performance, the first dummy *CutRD* is constructed to indicate whether managers cut R&D expenditure for pursuing higher performance, reversing earnings decline or reducing the scale of losses. Similarly, based on EPS performance, a second dummy *BeatbyCutRD* is constructed to indicate whether the management cuts R&D expenditure to beat analysts’ consensus forecasts. The construction process is explained in details as follows.

Regarding *CutRD*, this article follows Bushee (1998) and compares earnings before tax and R&D to its value from last year. This tells how the firm has performed in the short run. Specifically, we group sample companies into three categories according to this criterion. The first category includes firms with this year’s earnings before tax and R&D higher than that of last year (hence called the “increase” sub-sample). The second category includes firms with this year’s earnings before tax and R&D less than that of last year, but the difference turns out to be smaller than last year’s R&D expenditure (hence labeled the “small decrease” sub-sample). The third category consists of firms with this year’s earnings before tax and R&D less than that of last year, and the decrease turns out to be larger than last year’s R&D expenditure (hence referred to as the “large decrease” sub-sample). Intuitively speaking, managers of firms in the “small decrease” sub-sample have strong incentives to cut R&D expense to avoid earnings decline in the short term, while the myopic motivation in the “increase” and “large decrease” sub-sample are relatively weak. Therefore, a managerial myopia proxy, *CutRD*, equals one if the R&D expenditure in this year is smaller than last year (zero otherwise). Then the coefficients obtained by regressing *CutRD* on CEO locality in all three aforementioned sub-samples can imply whether non-local-province CEOs incline to reduce R&D expenditure for pursuing better short-term performance.

As for *BeatbyCutRD*, EPS is adopted as the short-term performance indicator and compared to analyst forecast. Kasznik and McNichols (2002) demonstrate that a company will have a valuation premium if it beats analyst forecast but will suffer stock price drop if its EPS falls short of analyst forecast. Specifically, for companies in its growing stage, the stock price fall will be sharper (Skinner and Sloan, 2002). Matsunaga and Park (2001) further provide evidence that a lower-than-analyst-forecast EPS harms the CEO’s annual bonus. Following Lai et al. (2020), *BeatByCutRD* equals 1 when the following two conditions are satisfied simultaneously; and 0 otherwise.


(1) $$\frac{E\,P\,S-E\,P\,S_{F\,o\,r\,e\,c\,a\,s\,t}}{C\,l\,o\,s\,e\,P\,r\,i\,c\,e}\geq 0;$$

(2) $$\frac{E\,P\,S-E\,P\,S_{F\,o\,r\,e\,c\,a\,s\,t}+\Delta \,R\&D}{C\,l\,o\,s\,e\,P\,r\,i\,c\,e}<0,$$

where *EPS*_*Forecast*_ represents the consensus forecast (proxied by the median analyst forecast) of EPS for the company of interest. Based on the Wind database, from which the analyst forecast data is obtained, the forecast period traces back to 180 days from the balance sheet date. ΔR&D is the yearly change of the R&D expenditure as a percentage of total equity. Therefore, if ΔR&D is less than 0, then the company must have reduced R&D expenditure during the year.

If only Equation (1) is satisfied, i.e., a firm’s earnings beat analysts’ consensus forecast, then it cannot be classified as suffering from managerial myopia yet. The reason is that the company may beat forecasts due to actual superior performance instead of cutting R&D expenditure. Thus, Equation (2) is a necessary condition to diagnose the existence of managerial myopia. As a result, when *BeatByCutRD* is equal to 1, the company beats analysts’ consensus forecasts by cutting R&D expenditure and would fall short of forecast if it does not cut R&D expenditure.

This article then utilizes three sets of Logit models to test the proposed hypotheses. Specifically, since the sample is panel data, a pooled, a random-effects, a fixed-effects, and a mixed-effects (partially fixed for province and for industry effects, respectively) logit regression is fit to the sample. The likelihood-ratio test does not reject a pooled model, and the 95% confidence interval for panel-level variance component contains zero for all specifications of (3)–(8). In addition, the insignificant level coefficients of mixed-effect models do not support province- or industry-specific effects. As for whether fixing the firm effects, Hausman test does not favor the fixed-effects model over the random-effects model in all cases. Therefore, a pooled regression model is employed to comprise for the short panel sample.

For hypothesis 1 that local-province CEOs are less myopic than non-local-province CEOs, it has:


(3) $$C\,u\,t\,R\,D_{i,t}=\alpha_{0}+\alpha_{1}\,L\,o\,c\,a\,l\,\_\,p\,r\,o\,v\,i\,n\,c\,e\,C\,E\,O_{i,t}+C\,o\,n\,t\,r\,o\,l\,s+\varepsilon_{i,t};$$

$$B\,e\,a\,t\,b\,y\,C\,u\,t\,R\,D_{i,t}=\beta_{0}+\beta_{1}\,L\,o\,c\,a\,l\,\_\,p\,r\,o\,v\,i\,n\,c\,e\,C\,E\,O_{i,t}$$

(4) $$+C\,o\,n\,t\,r\,o\,l\,s+\eta_{i,t},$$

where the key explanatory variable *Local-province CEO* is a dummy variable and equals 1 if the CEO’s native place or birthplace is in the same province as the company’s headquarters; and 0 otherwise. This variable is constructed at the province level for two reasons. On the one hand, when it is defined at the city or village level, the proportion of local CEOs is too low to yield effective regression results. On the other hand, people usually project their passions for hometown onto broader regions like provinces. Such projection is due to the unique image and identity of each province (Tuan, 1975). For instance, each province in China has its local dialects, folk customs, and eating habits.

*Controls* is a vector of control variables. Executive ownership is first to include since a greater proportion of executive owners makes the interests of the CEO more aligned with the interests of shareholders. This prompts CEOs to give priority to the long-term development of the company, mitigating the managerial myopia problem. Second, the change in capital expenditures is used to measure firm growth opportunities. An increasing expenditure change means the firm is in its expansion stage. Third, company size is controlled. Large companies draw a lot of public attention. Hence, investors are better informed, and the myopic behaviors of large-size firm CEOs would receive more intensive monitoring. The fourth control is leverage. Larger leverage implies more debt financing. The creditors also have incentives to supervise firm investment decisions by formulating covenants, which reduces the flexibility of management and space for earning manipulations. Fifth, Tobin’s *Q*, which is the ratio of a firm’s market value to its replacement cost, is included as a measurement of investment opportunities. Intuitively, if the market value is higher than the replacement cost, then the firm will conduct new projects via equity financing. Else if Tobin’s *Q* is less than 1, then investing in new projects would be non-profitable.

The sixth control is free cash flow, as managers are less likely to give up projects that can improve short-term performance if the company holds more cash. Seventh, institutional ownership is controlled. Institutions are sophisticated investors; hence can play important supervisory roles in the capital market. Bushee (1998) finds that, when a higher proportion of shares are owned by institutional investors, managers in the firm are less likely to reduce R&D expenses in exchange for reversing earnings decline. Second to the last, age should be considered. Lundstrum (2002) believes that firm R&D expenditures decrease with CEO’s age. Wu and Li (2012) also prove that CEO age is positively correlated with managerial myopia. Therefore, this article incorporates a retirement dummy, which equals one if the CEO’s age is greater than 63 years old; and 0 otherwise. Finally, Bertrand and Schoar (2003) provide evidence that the founder of a company often possesses a long horizon in the process of corporate decision making. Consequently, a founder identity dummy, which equals one if the CEO is also the founder of the company and 0 otherwise, is defined.

Now, it proceeds to introduce the specification to investigate mechanisms behind the CEO locality effect in China. To test the mediating effect of social capital on CEO locality effect in Hypothesis 2, an interaction term is included in the previous models and obtain:


$$C\,u\,t\,R\,D_{i,t}=\alpha_{0}+\alpha_{1}\,L\,o\,c\,a\,l\,\_\,p\,r\,o\,v\,i\,n\,c\,e\,C\,E\,O_{i,t}$$

$$+\alpha_{2}\,L\,o\,c\,a\,l\,\_\,p\,r\,o\,v\,i\,n\,c\,e\,C\,E\,O_{i,t}\times S\,o\,c\,i\,a\,l\,C\,a\,p\,i\,t\,a\,l_{i,t}$$

(5) $$+C\,o\,n\,t\,r\,o\,l\,s+\varepsilon_{i,t};$$

$$B\,e\,a\,t\,b\,y\,C\,u\,t\,R\,D_{i,t}=\beta_{0}+\beta_{1}\,L\,o\,c\,a\,l\,\_\,p\,r\,o\,v\,i\,n\,c\,e\,C\,E\,O_{i,t}$$

$$+\beta_{2}\,L\,o\,c\,a\,l\,\_\,p\,r\,o\,v\,i\,n\,c\,e\,C\,E\,O_{i,t}$$

(6) $$\times S\,o\,c\,i\,a\,l\,C\,a\,p\,i\,t\,a\,l_{i,t}+C\,o\,n\,t\,r\,o\,l\,s+\eta_{i,t},$$

where social capital is a mediating variable. To measure social capital, the literature provides several proxies. One is the number of associations and societies registered in a province (Rupasingha et al., 2006) since these organizations could facilitate the development of social networks. Another one is the degree of trust between people and the extent of citizen participation in provincial government decision-making. For example, Coleman (1988) uses the rate of migration across regions as a proxy for social capital. He argues that high population mobility leads to indirect connections, hence hindering social capital accumulation. Since China has instituted a migration policy designed to strictly control changes in permanent residence, this article employs the first proxy. Nevertheless, following Rupasingha et al. (2006), this article adopts a commonly used rating system, i.e., Report on Business Environment Index of Chinese Provinces (Wang et al., 2013, 2017), to proxy provincial social capital. This system assigns a score for each Chinese province based on the quality of associations and the number of societies registered within a province. A higher score implies more connections enabled by these organizations. A pleasant social environment promotes communications among members of the society, hence boosting the development of social capital.

To test the mediating effect of external formal institutions (e.g., formal arrangements by the government granting funds to support corporate innovation) on the CEO locality effect as stated in Hypothesis 3, the following regression equations are specified.


$$C\,u\,t\,R\,D_{i,t}=\alpha_{0}+\alpha_{1}\,L\,o\,c\,a\,l\,\_\,p\,r\,o\,v\,i\,n\,c\,e\,C\,E\,O_{i,t}$$

$$+\alpha_{2}\,L\,o\,c\,a\,l\,\_\,p\,r\,o\,v\,i\,n\,c\,e\,C\,E\,O_{i,t}\times G\,o\,v\,S\,u\,p\,p\,o\,r\,t_{i,t}$$

(7) $$+C\,o\,n\,t\,r\,o\,l\,s+\varepsilon_{i,t};$$

$$B\,e\,a\,t\,b\,y\,C\,u\,t\,R\,D_{i,t}=\beta_{0}+\beta_{1}\,L\,o\,c\,a\,l\,\_\,p\,r\,o\,v\,i\,n\,c\,e\,C\,E\,O_{i,t}$$

$$+\beta_{2}\,L\,o\,c\,a\,l\,\_\,p\,r\,o\,v\,i\,n\,c\,e\,C\,E\,O_{i,t}$$

(8) $$\times G\,o\,v\,S\,u\,p\,p\,o\,r\,t_{i,t}+C\,o\,n\,t\,r\,o\,l\,s+\eta_{i,t},$$

where government support is another mediating variable of interest. Shu and Hu (2017) summarize several common ways of supports, including government procurement, public finance, preferential tax privilege, the improved legal system, and strengthened infrastructure. This article defines the variable *GovSupport* as the proportion of R&D expenditure attributable to governmental funds.


$$G\,o\,v\,S\,u\,p\,p\,o\,r\,t=\frac{G\,o\,v\,e\,r\,n\,m\,e\,n\,t\,F\,u\,n\,d\,e\,d\,R\&D\,E\,x\,p\,e\,n\,d\,i\,t\,u\,r\,e\,s}{T\,o\,t\,a\,l\,F\,i\,r\,m\,R\&D\,E\,x\,p\,e\,n\,d\,i\,t\,u\,r\,e\,s}$$

Appendix Table A1 lists the definition of variables mentioned above.

## Results

### Descriptive statistics and preliminary analysis

Table 1 reports the summary statistics of the main variables. As can be seen, there are 46.6% of the company-year observations with decreased R&D expenditures, compared to 26.6% in the United States (Lai et al., 2020), implying cutting R&D is common in the sample period and companies in this article. The companies in the sample are also likely to transit between cutting or not cutting R&D expenditures, the probability for change from not cutting R&D expenditure to cutting R&D expenditure is 43.42%, and 57.62% vice versa. However, the cutting R&D behavior is not autocorrelated. About 10.8% (the mean of *BeatByCutRD*) of sample companies cut R&D expenditures to beat analysts’ consensus forecasts. This statistic for China is comparable to the estimate of 15% for the United States in Lai et al. (2020). The firm size is measured by the natural logarithm of total assets. The average size of sample companies is 22.08, corresponding to an asset amount of RMB 3.872 billion. Among all sample CEOs, 39.9% are founders of the companies. Only 3.3% (the mean of *Retirement*) of CEOs are approaching the retirement age threshold of 63. The distribution of local-province CEOs across year and province is illustrated in Appendix Table A2. Overall, 43.2% of sample firms hire locals as the CEO, and the proportion of local-province CEOs in Lai et al. (2020) sample is 21.3%. The large difference in the proportion of local-province CEOs between China’s and United States sample occurs for two reasons. First, this article determines local CEO according to the CEO’s native place or birthplace, while Lai et al. (2020) only use birthplace information. Secondly, the Hukou system in China prevents people from free migration, encouraging most of them to stay in their home province. Consequently, a higher proportion of local CEOs is observed in China. Appendix Table A3 presents between-group difference tests for the local-province and non-local-province group. The finding is that local-province CEOs serve significantly longer tenure but earn less salary than their non-local peers.

**TABLE 1:** Descriptive statistics.

Variable	*N*	Mean	SD	Min	P25	Median	P75	Max
*BeatByCutRD*	2,083	0.108	0.310	0	0	0	0	1
*CutRD*	2,083	0.464	0.499	0	0	0	1	1
*Local-province CEO*	2,083	0.432	0.495	0	0	0	1	1
*Executive ownership*	2,083	0.028	0.088	0	0	0	0	0.523
*Capital change*	2,083	0.104	0.780	−4.492	−0.316	0.085	0.504	5.086
*Size*	2,083	22.119	0.961	19.199	21.435	22.056	22.735	25.252
*Leverage*	2,083	0.057	0.066	0	0.008	0.028	0.089	0.444
*Tobin’s Q*	2,083	2.486	1.752	0.757	1.497	2.025	2.902	26.819
*Free cash flow*	2,083	−0.025	0.276	−3.412	−0.123	−0.007	0.094	3.322
*Capital intensity*	2,083	0.484	0.477	0.002	0.203	0.367	0.613	8.829
*Institutional ownership*	2,083	0.274	0.220	0	0.080	0.215	0.448	0.915
*Age*	2,083	49.828	6.438	30	46	50	54	74
*Retirement*	2,083	0.033	0.179	0	0	0	0	1
*Founder*	2,083	0.394	0.489	0	0	0	1	1
*Social capital*	2,075	3.144	0.318	2.410	2.89	3.11	3.38	3.900
*GovSupport*	2,083	0.048	0.085	0	0.018	0.025	0.055	0.863

The correlation coefficients are summarized in Appendix Table A4. The local-province CEO dummy is significantly and negatively correlated with the dependent variables. Variance inflation factor (VIF) is calculated to detect the severity of multicollinearity. The mean VIF is less than 2 according to the multicollinearity restuls in Appendix Table A5, indicating the multicollinearity is not problematic in the regressions. Finally, the likelihood ratio (LR) test is used to test heteroscedasticity. It turns out that the *p*-values of the LR test are much greater than 0.05. Therefore, the null hypothesis of homoscedasticity cannot be rejected, i.e., the regressions in this article do not suffer from heteroscedasticity problems.

### Baseline regression results

The baseline sample is a panel dataset. The effect of having a local-province CEO on managerial myopia in Chinese firms in the absence of mediating variables is first examined. Table 2 estimates (3) in which earnings are used as the short-term performance indicator.

**TABLE 2:** Local-province CEOs and managerial myopia (using earnings as the short-term performance indicator).

	(1)	(2)	(3)	(4)
Dep. var. is *CutRD*	Full sample	Subsample with increases in earnings before tax and R&D	Subsample with small decreases in earnings before tax and R&D	Subsample with large decreases in earnings before tax and R&D
*Local-province CEO*	−0.388***	−0.354***	−0.618***	−0.456
	(0.093)	(0.117)	(0.217)	(0.321)
*Executive ownership*	0.034	0.129	−1.165	3.424*
	(0.515)	(0.641)	(1.140)	(2.023)
*Capital change*	−0.198***	−0.028	−0.546***	−0.269
	(0.059)	(0.074)	(0.154)	(0.180)
*Size*	−0.123**	−0.246***	0.113	0.285
	(0.061)	(0.078)	(0.133)	(0.229)
*Leverage*	−1.041	−1.429	−1.540	1.917
	(0.769)	(0.975)	(1.726)	(2.625)
*Tobin’s Q*	0.056*	0.037	0.126	0.250*
	(0.030)	(0.036)	(0.080)	(0.150)
*Free cash flow*	−0.492***	−0.489**	0.126	−0.331
	(0.178)	(0.205)	(0.532)	(0.661)
*Capital intensity*	0.047	0.066	−0.003	0.121
	(0.101)	(0.119)	(0.281)	(0.338)
*Institutional ownership*	−0.228	−0.269	−0.166	−0.013
	(0.231)	(0.291)	(0.508)	(0.792)
*Retirement*	0.009	−0.115	0.514	−0.374
	(0.252)	(0.317)	(0.566)	(0.928)
*Founder*	0.042	0.002	0.172	0.028
	(0.098)	(0.122)	(0.213)	(0.330)
Intercept	2.698**	5.329***	−2.292	−6.744
	(1.362)	(1.724)	(3.004)	(5.120)
N	2,083	1,368	491	224
Pseudo *R*^2^	0.0237	0.0280	0.0466	0.0477

The sub-sample in column (2) includes firms with earnings before tax and R&D current year higher than last year. The sub-sample in column (3) includes firms with earnings before tax and R&D current year lower than last year, and the decrease turns out to be less than last year’s R&D expenditure. The sub-sample in column (4) further narrows the column (3) subsample down to include only firms with earnings decreasing more than the last year’s R&D expenditure. All results are estimated using the logistic regression. ***, **, and * indicate significance at the 1%, 5%, and 10% level, respectively. The values in the parentheses are standard errors.

Column (2) of Table 2 reports the results of regressing in the “increase” subsample. The coefficient of *Local-province CEO* is negative and statistically significant, meaning that non-local-province CEOs’ propensity to cut R&D expenditures for pursuing higher earnings, even when the earnings increase and the pressure for short-term performance is relatively low. In particular, the probability of local-province CEOs exchanging R&D expenses for immediate profits is 8.33% lower than that of non-local-province CEOs, holding other variables constant. This result based on the Chinese sample differs from that in Lai et al. (2020) who study the United States sample. The coefficient of local-state CEO is negative but non-significant—no significant difference in myopic behaviors between local-state and non-local state CEOs—in the “increase” subsample. Such interesting divergence between the Chinese sample and the United States sample can be attributed to the differences in the executive incentive system between two the countries. The Chinese system lacks long-term incentive arrangements because the capital markets in China as the foundation of all kinds of incentive mechanisms are still at their growing stage. Moreover, executive compensation in Chinese private-sector firms is positively associated with short-term performance indicators such as ROA (Wu and Wu, 2010). Consequently, non-local-province CEOs in China are more motivated to maximizing their income and secure their bonus, when a firm has increased profit, no matter the size of the increase. In general, non-local CEOs ignore firm long-term investments.

Executive stockholding is expected as an effective means to mitigate managerial myopia. However, the coefficient of *Executive ownership* is positive but non-significant in the “increase” subsample. Abdullah et al. (2002) prove the existence of a W-shaped relation between R&D expenditures and executives’ shareholding. If executives hold only 0–5% shares, then the ratio would be too low for interest alignment to take effect. On the opposite, such a low ratio can cause myopic behaviors, leading to a negative association between R&D expenditures and managerial shares. When the shareholding ratio is higher than 15%, the interests of managers overlap with those of owners, resulting in a strong positive correlation between R&D expenditures and manager stockholding. The 17.47% of the observations in this group have non-zero *Executive ownership*, and the mean of *Executive ownership* is 0.0284, which falls in the range of 0–5%. A similar phenomenon is observed in China by Tang and Yi (2010)—although executive shareholding can stimulate R&D investments, the stimulating effect is ignorable if the proportion of shares held by executives fall below a threshold of 0.1%. Hence, the non-significant coefficient for *Executive ownership* is consistent with the findings in the literature.

The negative coefficient of *Size* indicates that bigger companies have longer investment horizons. The reason is that big companies receive great public attention, and investors hence can obtain more information and effectively monitor manager myopia so that such behaviors are deterred in the very beginning. *Tobin’s Q* has a positive but non-significant coefficient. A higher *Tobin’s Q* indicates that new investment will be profitable. Thus, CEOs must make a tradeoff between long- and short-term projects.

The negative coefficient of *Free cash flow* suggests that, given sufficient cash flows for a public firm, its managers will balance long- and short-term investments more properly. *Retirement* has a negative but non-significant coefficient, probably due to the small proportion of CEOs close to retirement in the sample. The coefficient of *Founder* is non-significant and positive, which is contrary to the findings with United States companies in Lai et al. (2020) and Schuster et al. (2020). It is believed that the labor market for professional managers plays a role here. In the United States sample of Lai et al. (2020), only 7.5% of the CEOs are also founders of the companies. In this sample, 39.4% of Chinese firms appoint founders as executives. Besides, the founders of publicly listed private-sector companies are likely to embrace short-termism due to their educational background and experiences (Wu, 2001).

Table 2 column (3) estimates Equation (3) for the “small decrease” subsample. This is the group facing the largest short-term performance pressure among the three groups. The coefficient of *Local-province CEO* is statistically significant and negative as expected. That is, non-local-province CEOs are 13.88% more likely to reduce R&D expenses for avoiding earnings decline than local-province CEOs. This finding is consistent with Lai et al. (2020). It is noteworthy that as CEOs in the “small decrease” group have a stronger incentive to prevent a decline in earnings than other groups, the coefficient of *Local-province CEO* is unsurprisingly the largest in absolute-value magnitude across all columns in Table 2. The coefficient of *Executive ownership* is still not significant. The 84.11% of observations in this subsample have *Executive ownership* of value 0, and the average executive shareholding percentage is 2.77%. The negative and significant coefficient of *Capital change* suggests that when the company is in the stage of rapid expansion, its managers are unlikely to behave myopically. This is because the opportunity costs of myopia are high for them.

Table 2 column (4) reports the results from the “large decrease” subsample. In this group, the earnings decline is expected to be so large that it cannot be reversed by cutting R&D investments by last year’s total R&D expenses. In this case, local-province and non-local-province CEOs will behave similarly in terms of myopia. The coefficient of *Local-province CEO* is now non-significant. It is interesting to find the coefficient for *Executive ownership* is positive and weakly significant. The 12.5% of 224 observations in this group have *Executive ownership* of value 0, and the average executive shareholding percentage is 2.52%. The estimate may be biased by such a small proportion of executive ownership.

To sum up, non-local-province CEOs are prone to forego R&D for avoiding earnings decline (whenever possible) or pursuing higher earnings in comparison to local-province CEOs.

Table 3 reports the regression results of model (4). The coefficient of *Local-province CEO* is again negative and statistically and economically significant. The likelihood of local-province CEOs reducing R&D expenditures for beating analysts’ consensus forecasts is 4.72% lower than that of non-locals. The coefficient of *Executive ownership* is not statistically significant. The 85.6% of observations in the overall sample have *Executive ownership* equaling 0, and the average executive shareholding percentage is 2.8%. In conclusion, local-province CEOs in China are less subject to myopic behaviors.

**TABLE 3:** Local CEOs and managerial myopia (using EPS as the short-term performance indicator).

Dep. var. is *BeatByCutRD*	Full sample
*Local-province CEO*	−0.500***
	(0.155)
*Executive ownership*	0.061
	(0.800)
*Capital change*	0.202**
	(0.088)
*Size*	−0.014
	(0.097)
*Leverage*	−1.805
	(1.310)
*Tobin’s Q*	0.079**
	(0.036)
*Free cash flow*	−0.360
	(0.251)
*Capital intensity*	0.100
	(0.141)
*Institutional ownership*	−0.273
	(0.379)
*Retirement*	−0.471
	(0.475)
*Founder*	0.155
	(0.154)
Intercept	−1.793
	(2.132)
*N*	2,083
Pseudo *R*^2^	0.0246

All results are estimated using the logistic regression. ***, **, and * indicate significance at the 1%, 5%, and 10% level, respectively. The values in the parentheses are standard errors.

In what follows, the mediating effects of social capital and of government R&D subsidy on the relationship between local CEO and the myopic behaviors will be discussed. Concerning the effect of social capital, Table 4 reports estimation results of specification (5) with observations in the “increase” and “small decrease” subsample, as managerial myopia is not observed in the “large decrease” subsample. The coefficient of *Local-province CEO* turns significantly positive after the provincial social capital and the interaction term are included in the model. It implies that if social capital is controlled, local CEOs have higher chance to behave myopically than their non-local peers. Unlike the results in Lai et al. (2020), where the coefficient of *Local-province CEO* is no longer significant after controlling for social capital. The significantly negative coefficient of *Social capital* confirms that social capital contributes to reducing the myopic behavior of non-local CEOs. The significant negative coefficients of the interaction item, *Local-province CEO* × *Social capital* imply that when a province has a social capital rating higher than 2.42 (the minimum of *Social capital* in the sample of this article is 2.41), the myopic behavior of local CEO will be reverted. Thus, the locality effect on managerial myopia is more prominent if a province has larger social capital, and the mediating effect of social capital is more salient in China than in the United States.

**TABLE 4:** Social capital, local-province CEOs, and managerial myopia.

	(1)	(2)
Dep. var. is *CutRD* for column (1); and *BeatByCutRD* for column (2)	Subsample with increases or small decreases in earnings before tax and R&D	Full sample
*Local-province CEO*	1.486	7.643***
	(0.981)	(1.747)
*Social capital*	−0.502**	−0.402
	(0.214)	(0.298)
*Local-province CEO* × *Social capital*	−0.615**	−2.752***
	(0.313)	(0.590)
*Executive ownership*	−0.227	0.083
	(0.552)	(0.798)
*Capital change*	−0.146**	0.228**
	(0.065)	(0.090)
*Size*	−0.124*	0.044
	(0.065)	(0.098)
*Leverage*	−1.654**	−1.721
	(0.839)	(1.338)
*Tobin’s Q*	0.037	0.074**
	(0.031)	(0.037)
*Free cash flow*	−0.510***	−0.348
	(0.189)	(0.252)
*Capital intensity*	0.000	0.066
	(0.108)	(0.143)
*Institutional ownership*	−0.304	−0.429
	(0.251)	(0.383)
*Retirement*	0.045	−0.478
	(0.270)	(0.482)
*Founder*	0.035	0.144
	(0.105)	(0.157)
Intercept	4.396***	−1.737
	(1.573)	(2.301)
*N*	1,851	2,075
Pseudo *R*^2^	0.0363	0.0613

The sub-sample in column (1) includes two groups of firms. The first group consists of firms with earnings before tax and R&D current year higher than last year. The second group consists of firms with earnings before tax and R&D current year lower than last year, and the decrease turns out to be less than last year’s R&D expenditure. All results are estimated using the logistic regression. ***, **, and * indicate significance at the 1%, 5%, and 10% level, respectively. The values in the parentheses are standard errors.

The effect of government subsidy on enterprise’s innovation is controversial. Table 5 reports estimation results of Equation (7) with the “increase” and “small decrease” subsample. The significant positive coefficients of the interaction item, *Local-province CEO* × *GovSupport*, suggests that the CEO locality effect will be weakened if the company receives more government financial funds for innovation. The coefficient for *GovSupport* is insignificant though, suggesting that if the company hires non-local-province CEO, the government subsidy has no significant effect on the company’s innovation activity. This result is consistent with the findings in the literature, that the effect of government subsidy for innovation is ambiguous (e.g., inverted U-shaped) in China (Yi et al., 2021; Xia et al., 2022). Since the 75th percentile of *GovSupport* in the sample is 0.055, at least 75% of the companies receive a quite low level of government financial support, and the locality effect on the managerial myopic behaviors is still significantly negative (−0.323 given the government subsidy that the company receives accounts for 5.5% of its total R&D expenditure). Once the subsidy is above 10.46% of a firm’s total R&D expenditure, and the firm has a local CEO, the CEO may be more myopic than her or his non-local peers and the firm may have a higher chance to cut its R&D expenditure even when its profit increases. It seems that a low level of government subsidy mitigates the discrepancy in myopic behavior between local and non-local CEOs, while a higher level of subsidy may revert the behavior. This is quite different to the findings of Le and Jaffe (2016) from New Zealand firms, that compared to smaller, non-project-specific government grants, larger, project-based grants are more effective at promoting innovation. It merits a last note that we have low values of pseudo *R*^2^’s in our tables as in many psychology studies, suggesting the unpredictability of myopia behaviors and a large number of potential determinants of managerial myopia. Nevertheless, since *R*^2^ is a measure of the whole model rather than the significance of relation between the dependent variable and the key independent variable, which is the focus of this article, it can be safely concluded that our proposed CEO locality serves as a significant myopia predictor, although managerial myopia contains an inherently higher amount of unexplainable variability.

**TABLE 5:** Government support, local-province CEOs, and managerial myopia.

	(1)	(2)
Dep. var. is *CutRD* for column (1); and *BeatByCutRD* for column (2)	Subsample with increases or small decreases in earnings before tax and R&D	Full sample
*Local-province CEO*	−0.681***	−1.286***
	(0.139)	(0.231)
*GovSupport*	0.446	2.783
	(1.681)	(1.967)
*Local-province CEO* × *GovSupport*	6.512**	8.630***
	(2.530)	(3.101)
*Executive ownership*	−0.298	0.066
	(0.559)	(0.870)
*Capital change*	−0.168***	0.209**
	(0.065)	(0.093)
*Size*	−0.148**	0.005
	(0.066)	(0.103)
*Leverage*	−1.737**	−2.211
	(0.841)	(1.416)
*Tobin’s Q*	0.042	0.074*
	(0.032)	(0.038)
*Free cash flow*	−0.460**	−0.306
	(0.189)	(0.262)
*Capital intensity*	0.078	0.172
	(0.109)	(0.146)
*Institutional ownership*	−0.228	−0.291
	(0.251)	(0.413)
*Retirement*	0.061	−0.482
	(0.269)	(0.528)
*Founder*	0.037	0.144
	(0.105)	(0.166)
Intercept	3.257**	−2.337
	(1.460)	(2.269)
*N*	1,859	2,083
Pseudo *R*^2^	0.0437	0.1338

The sub-sample in column (1) includes two groups of firms. The first group consists of firms with earnings before tax and R&D current year higher than last year. The second group consists of firms with earnings before tax and R&D current year lower than last year, and the decrease turns out to be less than last year’s R&D expenditure. All results are estimated using the logistic regression. ***, **, and * indicate significance at the 1%, 5%, and 10% level, respectively. The values in the parentheses are standard errors.

### Further tests and discussion

This section discusses the robustness of the results. First, the endogeneity problem caused by omitted variables has already been dealt with. As suggested by relevant studies, this article includes executive ownership, change in capital, firm size, leverage ratio, Tobin’s *Q*, free cash flow, capital intensity, and institutional ownership to control for the sample company’s financial positions and corporate governance quality. Besides, this article also adds CEO retirement status and founder or not to account for the effect of CEOs’ personal information on their myopic behaviors. The results stay unchanged before and after incorporating these controls.

Second, this article focuses on potential endogeneity due to reverse causality. As the mechanism of the CEO locality effect lies in people’s hometown attachment or government’s innovation support, both reputation pressure and subsidy demand may, in turn, affect the hiring decisions of the firm committee. Moreover, local-province CEOs themselves may choose to work for companies that exhibit their preference for long-term benefits. Because these CEOs believe that their utility can be maximized in companies that share common interests with them. As a result, this article resorts to the following two methods in isolating the desired direction of the causal relationship in the baseline model.

One method is the DID approach. Dechow and Sloan (1991) find that a company spends significantly less on R&D activities when the CEO is in her last year of office. Rao et al. (2012) discover that there exists an inverted U-shape relation between CEOs’ phase of tenure and their investment horizon. Specifically, CEOs in the early and late years of their term are likely to act myopically, and CEOs in their mid-career have more long-term thinking. Based on these findings, this article treats CEOs’ last year of office as a shock and utilizes the DID estimation to verify how being in the last year influence the CEO locality effect in Chinese firms. The two conditions for performing DID analysis are satisfied here. On the one hand, CEOs’ last year of office is an exogenous shock. On the other hand, the sample covers at least 1 year’s panel data before and after the implementation of this shock. A new variable *Last year* is defined, and its value equals one when the observation year is the CEO’s last year of office, and 0 otherwise. The model is specified as follows.


$$C\,u\,t\,R\,D_{i,t}=\alpha_{0}+\alpha_{1}\,L\,o\,c\,a\,l\,\_\,p\,r\,o\,v\,i\,n\,c\,e\,C\,E\,O_{i,t}$$

$$+\alpha_{2}\,L\,o\,c\,a\,l\,\_\,p\,r\,o\,v\,i\,n\,c\,e\,C\,E\,O_{i,t}\times L\,a\,s\,t\,y\,e\,a\,r_{i,t}$$

(9) $$+C\,o\,n\,t\,r\,o\,l\,s+\varepsilon_{i,t};$$

$$B\,e\,a\,t\,b\,y\,C\,u\,t\,R\,D_{i,t}=\beta_{0}+\beta_{1}\,L\,o\,c\,a\,l\,\_\,p\,r\,o\,v\,i\,n\,c\,e\,C\,E\,O_{i,t}$$

$$+\beta_{2}\,L\,o\,c\,a\,l\,\_\,p\,r\,o\,v\,i\,n\,c\,e\,C\,E\,O_{i,t}\times L\,a\,s\,t\,y\,e\,a\,r_{i,t}$$

(10) $$+C\,o\,n\,t\,r\,o\,l\,s+\eta_{i,t},$$

Table 6 summarizes the DID results. In column (1), the coefficient of *Local-province CEO* × *Last year* is −0.934, and is significant, implying that, in CEOs’ last year of office, non-local-province CEOs are more inclined to reduce R&D expenses than local-province CEOs. In column (2), the coefficient of the interaction term is not significant since CEOs’ salary in their last tenure year is insensitive to analyst forecasts. Gibbons and Murphy (1992) state that CEO compensation is based on historical performance rather than analyst forecasts when executives are getting closer to the last day of their services in the office.

**TABLE 6:** Last year of office, local-province CEOs, and managerial myopia.

	(1)	(2)
Dep. var. is *CutRD* for column (1), and *BeatByCutRD* for column (2)	Full sample	Full sample
*Local-province CEO*	−0.244**	−0.478***
	(0.100)	(0.161)
*Last year of office*	0.714***	−0.000
	(0.165)	(0.004)
*Local-province CEO* × *Last year of office*	−0.934***	−0.547
	(0.259)	(0.424)
*Executive ownership*	0.065	0.063
	(0.518)	(0.802)
*Capital change*	−0.182***	0.205**
	(0.060)	(0.089)
*Size*	−0.130**	−0.007
	(0.061)	(0.097)
*Leverage*	−1.202	−1.993
	(0.776)	(1.321)
*Tobin’s Q*	0.054*	0.079**
	(0.030)	(0.037)
*Free cash flow*	−0.475***	−0.404
	(0.178)	(0.250)
*Capital intensity*	0.080	0.173
	(0.101)	(0.142)
*Institutional ownership*	−0.211	−0.264
	(0.232)	(0.379)
*Retirement*	−0.103	−0.810
	(0.257)	(0.544)
*Founder*	0.070	0.152
	(0.098)	(0.154)
Intercept	2.705**	−1.961
	(1.368)	(2.135)
*N*	2,082	2,082
Pseudo *R*^2^	0.0302	0.0285

The variable “last year of office” is a dummy that equals one if the observation year happens to be the concerned CEO’s last year of office, and 0 otherwise. All results are estimated using the logistic regression. ***, **, and * indicate significance at the 1%, 5%, and 10% level, respectively. The values in the parentheses are standard errors.

In addition to DID, another method that can mitigate the two-way causality problem is restricting the sample to firms that have changed their managers from local-province CEOs to non-local-province CEOs or the other way around. The logic behind this exercise is as follows. Given that corporate culture and strategies are relatively stable, if the change of firm myopic behaviors is observed when a company changes its local-province CEO to a non-local-province one or vice versa, then the direction of causal relationship can be verified. Thus, a sample is constructed by only including companies that had at least one CEO turnover. A collection of new variables are created accordingly. First, Δ*Local-province CEO* describes the change of CEO locality, which equals one if the firm has a local-province CEO to replace a non-local-province one; negative one if a non-local-province CEO succeed a local-province CEO, and 0 otherwise. The 70.61% of the values of Δ*Local-province CEO* in the CEO turnover sample is zero. The dummy *AdjCutRD* equals to one for a firm if (i) R&D expenditures in this year are lower than those of last year; and (ii) this year’s earnings before tax and R&D are higher than last year’s, or this year’s earnings before tax and R&D is less than last year’s but the drop is less than last year’s R&D expenditures. Next, Δ*AdjCutRD* is defined as the difference in the average of *AdjCutRD* between two successive CEOs. Other variables with a Δ notation are defined similarly.

Table 7 contains results with the CEO turnover sample. In column (1), the negative and significant coefficient of *Local-province CEO* suggests that when the local-province CEO replaces the non-local-province CEO, the company’s tendency to reduce R&D expenditures for attaining higher performance or avoiding earnings decline becomes significantly weaker. The same coefficient in column (2) turns non-significant because the percentage of companies with *BeatByCutRD* = 1 is much smaller than that with *CutRD* = 1 in the whole sample and even smaller in the CEO turnover sample.

**TABLE 7:** CEO turnover analysis.

	(1)	(2)
Dep. var. is Δ *AdjCutRD* for column (1), and Δ *BeatByCutRD* for column (2)	Full sample	Full sample
Δ*Local-province CEO*	−0.397***	−0.061
	(0.058)	(0.040)
Δ*Executive ownership*	−0.345	−0.088
	(0.625)	(0.430)
Δ*Capital change*	0.007	0.077***
	(0.038)	(0.026)
Δ*Size*	−0.005	−0.016
	(0.056)	(0.038)
Δ*Leverage*	0.508	−0.420
	(0.572)	(0.393)
Δ*Tobin’s Q*	−0.013	0.029
	(0.029)	(0.020)
Δ*Free cash flow*	−0.019	0.015
	(0.095)	(0.065)
Δ*Capital intensity*	0.078	0.068
	(0.063)	(0.044)
Δ*Institutional ownership*	0.268	0.006
	(0.206)	(0.142)
Δ*Retirement*	−0.308	−0.078
	(0.319)	(0.220)
Δ*Founder*	0.065	0.031
	(0.097)	(0.067)
Intercept	−0.182***	0.001
	(0.036)	(0.025)
*N*	228	228
*R* ^2^	0.198	0.082

This table presents the results of using CEO turnover in the same company to investigate the causal relationship between whether the CEO is from the local province and the associated managerial myopia behaviors. All results are estimated by the ordinary least squares regression. ***, **, and * indicate significance at the 1%, 5%, and 10% level, respectively. The values in the parentheses are standard errors.

As for the third robustness test, the current independent variable is substituted by its one-period lagged alternative. If CEO turnover does not occur during this year, then the pattern of myopic behaviors will probably stay unchanged. But if the CEO of a company this year is not the same person as in the last year, then the new CEO needs some time to form her management style and adjust firm strategies after she is in charge. Under these circumstances, the management style left by the previous CEO may still exert influences on the decision-making process of the company. Tables 8, 9 report the regression results with lagged main explanatory variables. The results show that if the CEO from the last year is a local in the firm’s headquarters province, then the probability of the company to cut R&D expenditures will be lower this year. Therefore, the findings of this article are robust.

**TABLE 8:** Robustness test: Replacing the independent variable (local-province CEOs and managerial myopia).

	(1)	(2)	(3)	(4)	(5)
Dep. var. is *CutRD* for columns (1)–(4), and *BeatByCutRD* for column (5)	Full sample	Subsample with increases in earnings before tax and R&D	Subsample with small decreases in earnings before tax and R&D	Subsample with large decreases in earnings before tax and R&D	Full sample
*Lagged local-province CEO*	−0.276***	−0.256**	−0.444**	−0.264	−0.468***
	(0.092)	(0.115)	(0.206)	(0.312)	(0.153)
*Executive ownership*	0.027	0.117	−1.124	3.334*	0.059
	(0.514)	(0.640)	(1.120)	(2.019)	(0.799)
*Capital change*	−0.193***	−0.024	−0.529***	−0.272	0.206**
	(0.059)	(0.074)	(0.151)	(0.181)	(0.088)
*Size*	−0.121**	−0.243***	0.113	0.271	−0.013
	(0.061)	(0.077)	(0.131)	(0.227)	(0.097)
*Leverage*	−0.995	−1.379	−1.530	2.106	−1.827
	(0.767)	(0.973)	(1.697)	(2.622)	(1.313)
*Tobin’s Q*	0.059*	0.039	0.130	0.247*	0.080**
	(0.030)	(0.036)	(0.080)	(0.148)	(0.036)
*Free cash flow*	−0.507***	−0.493**	0.027	−0.392	−0.380
	(0.177)	(0.204)	(0.522)	(0.660)	(0.250)
*Capital intensity*	0.045	0.068	−0.037	0.117	0.120
	(0.100)	(0.118)	(0.276)	(0.338)	(0.141)
*Institutional ownership*	−0.253	−0.310	−0.138	0.019	−0.275
	(0.231)	(0.290)	(0.502)	(0.790)	(0.379)
*Retirement*	−0.002	−0.119	0.480	−0.441	−0.479
	(0.252)	(0.316)	(0.557)	(0.921)	(0.475)
*Founder*	0.013	−0.026	0.136	−0.005	0.135
	(0.097)	(0.121)	(0.209)	(0.328)	(0.153)
Intercept	2.605*	5.226***	−2.375	−6.525	−1.837
	(1.360)	(1.720)	(2.960)	(5.090)	(2.133)
N	2,083	1,368	491	224	2,083
Pseudo *R*^2^	0.0196	0.0257	0.0403	0.0431	0.0237

All results are estimated using the logistic regression. ***, **, and * indicate significance at the 1%, 5%, and 10% level, respectively. The values in the parentheses are standard errors.

**TABLE 9:** Robustness test: Replacing the independent variable (social capital, GovSupport, and the CEO locality effect).

	(1)	(2)	(3)	(4)
Dep. var. is *CutRD* for columns (1)–(2), and *BeatByCutRD* for columns (3)–(4)	Subsample with increases or small decreases in earnings before tax and R&D		Full sample	
*Lagged local-province CEO*	1.651	−0.603***	5.334***	−1.774***
	(1.080)	(0.154)	(1.773)	(0.283)
*Social capital*	−0.368		−0.570*	
	(0.236)		(0.326)	
*Lagged local-province CEO* × *Social capital*	−0.605*		−1.939***	
	(0.339)		(0.584)	
*GovSupport*		−0.811		−0.816
		(2.190)		(3.117)
*Lagged local-province CEO* × *GovSupport*		7.900***		13.887***
		(2.860)		(4.101)
*Executive ownership*	−0.710	−0.919	−1.164	−2.578*
	(0.650)	(0.668)	(1.127)	(1.444)
*Capital change*	−0.115*	−0.156**	0.258***	0.217**
	(0.069)	(0.070)	(0.096)	(0.104)
*Size*	−0.130*	−0.132*	−0.005	0.017
	(0.071)	(0.072)	(0.107)	(0.116)
*Leverage*	−1.873**	−1.929**	−1.568	−2.075
	(0.900)	(0.907)	(1.437)	(1.570)
*Tobin’s Q*	0.033	0.044	0.071*	0.084**
	(0.033)	(0.033)	(0.039)	(0.040)
*Free cash flow*	−0.428**	−0.369*	−0.498*	−0.419
	(0.201)	(0.200)	(0.286)	(0.305)
*Capital intensity*	−0.020	0.041	0.072	0.198
	(0.111)	(0.111)	(0.152)	(0.159)
*Institutional ownership*	−0.497*	−0.444	−0.308	−0.361
	(0.274)	(0.276)	(0.416)	(0.468)
*Retirement*	−0.014	0.003	−0.647	−0.655
	(0.281)	(0.284)	(0.534)	(0.610)
*Founder*	−0.052	−0.043	0.131	0.162
	(0.112)	(0.113)	(0.169)	(0.185)
Intercept	4.132**	2.976*	−0.135	−2.434
	(1.773)	(1.605)	(2.596)	(2.558)
N	1,851	1,859	2,075	2,083
Pseudo *R*^2^	0.0297	0.0463	0.0591	0.1842

All results are estimated using the logistic regression. ***, **, and * indicate significance at the 1%, 5%, and 10% level, respectively. The values in the parentheses are standard errors.

At last, robustness is checked by excluding some special provinces from the baseline sample. Appendix Table A2 shows that the number of companies in some provinces is relatively low, which may affect the regression results. Therefore, the data of provinces with the number of observations less than ten is excluded and the regression models are estimated again. Tables 10, 11 summarize the corresponding results, and the main conclusion of this article remain unchanged.

**TABLE 10:** Robustness test: Excluding sample firms from certain provinces (local-province CEOs and managerial myopia).

	(1)	(2)	(3)	(4)	(5)
Dep. var. is *CutRD* for columns (1)–(4), and *BeatByCutRD* for column (5)	Full sample	Subsample with increases in earnings before tax and R&D	Subsample with small decreases in earnings before tax and R&D	Subsample with large decreases in earnings before tax and R&D	Full sample
*Local-province CEO*	−0.402***	−0.373***	−0.643***	−0.406	−0.505***
	(0.094)	(0.118)	(0.218)	(0.330)	(0.156)
*Executive ownership*	−0.016	0.047	−1.145	3.570*	0.098
	(0.518)	(0.646)	(1.134)	(2.071)	(0.801)
*Capital change*	−0.193***	−0.024	−0.520***	−0.263	0.207**
	(0.060)	(0.075)	(0.154)	(0.184)	(0.089)
*Size*	−0.127**	−0.253***	0.116	0.240	−0.018
	(0.062)	(0.079)	(0.134)	(0.237)	(0.097)
*Leverage*	−0.996	−1.444	−1.353	2.119	−1.546
	(0.776)	(0.986)	(1.735)	(2.720)	(1.310)
*Tobin’s Q*	0.057*	0.040	0.126	0.226	0.082**
	(0.031)	(0.037)	(0.080)	(0.155)	(0.037)
*Free cash flow*	−0.468***	−0.491**	0.248	−0.273	−0.337
	(0.179)	(0.206)	(0.546)	(0.674)	(0.253)
*Capital intensity*	0.041	0.035	0.084	0.200	0.110
	(0.103)	(0.121)	(0.288)	(0.369)	(0.144)
*Institutional ownership*	−0.200	−0.230	−0.155	0.150	−0.232
	(0.235)	(0.295)	(0.514)	(0.827)	(0.383)
*Retirement*	0.058	−0.011	0.487	−0.780	−0.376
	(0.258)	(0.322)	(0.565)	(1.073)	(0.476)
*Founder*	0.049	0.019	0.162	0.052	0.166
	(0.098)	(0.122)	(0.214)	(0.342)	(0.155)
Intercept	2.765**	5.479***	−2.385	−5.825	−1.756
	(1.377)	(1.750)	(3.006)	(5.310)	(2.151)
*N*	2,043	1,342	481	220	2,043
Pseudo *R*^2^	0.0227	0.0290	0.0459	0.0466	0.0245

All results are estimated using the logistic regression. ***, **, and * indicate significance at the 1%, 5%, and 10% level, respectively. The values in the parentheses are standard errors.

**TABLE 11:** Robustness test: Excluding sample firms from certain provinces (social capital, GovSupport, and the CEO locality effect).

	(1)	(2)	(3)	(4)
Dep. var. is *CutRD* for columns (1)–(2), and *BeatByCutRD* for columns (3)–(4)	Subsample with increases or small decreases in earnings before tax and R&D		Full sample	
*Local-province CEO*	1.358	−0.681***	7.512***	−1.281***
	(0.991)	(0.139)	(1.761)	(0.231)
*Social capital*	−0.535**		−0.435	
	(0.216)		(0.302)	
*Local-province CEO* × *Social capital*	−0.579*		−2.704***	
	(0.316)		(0.594)	
*GovSupport*		0.742		2.830
		(1.674)		(1.975)
*Local-province CEO* × *GovSupport*		6.075**		8.379***
		(2.495)		(3.047)
*Executive ownership*	−0.310	−0.370	0.103	0.106
	(0.556)	(0.563)	(0.800)	(0.872)
*Capital change*	−0.144**	−0.165**	0.235***	0.214**
	(0.065)	(0.065)	(0.090)	(0.093)
*Size*	−0.121*	−0.149**	0.041	0.002
	(0.066)	(0.066)	(0.099)	(0.104)
*Leverage*	−1.620*	−1.724**	−1.576	−1.923
	(0.846)	(0.850)	(1.343)	(1.417)
*Tobin’s Q*	0.040	0.045	0.078**	0.077**
	(0.032)	(0.032)	(0.037)	(0.038)
*Free cash flow*	−0.481**	−0.449**	−0.340	−0.287
	(0.190)	(0.190)	(0.254)	(0.265)
*Capital intensity*	−0.001	0.067	0.082	0.179
	(0.109)	(0.110)	(0.146)	(0.148)
*Institutional ownership*	−0.320	−0.200	−0.407	−0.243
	(0.253)	(0.255)	(0.385)	(0.418)
*Retirement*	0.122	0.145	−0.393	−0.362
	(0.274)	(0.273)	(0.484)	(0.528)
*Founder*	0.040	0.045	0.151	0.156
	(0.105)	(0.106)	(0.158)	(0.167)
Intercept	4.431***	−1.616	3.266**	−2.301
	(1.589)	(2.321)	(1.477)	(2.290)
*N*	1,823	2,043	1,823	2,043
Pseudo *R*^2^	0.0370	0.0445	0.0615	0.1352

All results are estimated using the logistic regression. ***, **, and * indicate significance at the 1%, 5%, and 10% level, respectively. The values in the parentheses are standard errors.

The above baseline and robustness findings confirm that Chinese local CEOs indeed behave less myopically than that of non-local CEOs. In specific, those local CEOs are less likely to cut R&D expenditures for beating analyst forecasts or avoiding earnings decreases, just like what has been discovered by previous studies employing the United States firm sample (e.g., Lai et al., 2020). However, this article differs from those similar studies in identifying a distinct mechanism underlying the CEO locality effect in China. While they suggest that the CEO locality effect is more significant in regions with high social capital in the developed world, this study documents that in emerging market economies, besides this informal social bond, formal government intervention also play a crucial role in the CEO locality effect.

## Conclusion

Short-term performance often outweighs long-term interest for self-interested managers. Managerial myopia occurs when this becomes normality. Managerial myopia hurts the development of firms in the long run and induces the management to deviate from the goal of maximizing firm value for shareholders. This article investigates whether local-province CEOs (the CEO’s native place or birthplace is in the same province as his company’s headquarters) are less subject to myopic behaviors than non-local-province CEOs in China based on financial and R&D data of all Chinese private-sector public companies during the period of 2014–2018. The results find that, setting local-province CEOs as the benchmark, non-local-province CEOs have a higher propensity to reduce R&D expenditures for beating analysts’ consensus forecasts, avoiding earnings decline, or pursuing higher earnings. It is also demonstrated that this so-called CEO locality effect is strengthened and weakened by, respectively, larger provincial social capital and more local-governmental subsidy. In more details, it is the social capital that twists the originally myopic propensity of local CEOs, but a higher level of government subsidy may increase a firm’s dependency on subsidy and motivate local CEOs to cut R&D expenditure and pursue short-term goals. The causality running from CEOs’ native place to managerial myopia is identified by a DID setup with CEO turnover. All findings stay robust to a variety of validations.

The theoretical significance of this article is twofold. First, the previous literature mainly focuses on formal solutions to managerial myopia, and few scholars explore the influential factors from the perspective of informal institutions. As an extension of Lai et al. (2020), this article deals distinctive evidence in the difference of myopic behaviors between non-local-province CEOs and the local-province CEOs in China, and in general providing new empirical evidence for the field of managerial myopia. Second, when formal institutions are improved, informal institutions such as companies hiring locals as CEOs no longer play an effective role in mitigating managerial myopia. However, the extent of the role the formal institutions play is tricky. The conclusion in this article reflects the complementary relationship between formal institutions and informal institutions.

The practical significance of this article lies in three aspects. First, formal institutions such as equity ownership, employment contract, and severance contracts can mitigate short-termism, but there are financial costs if companies grant stocks to CEOs or sign such contracts with CEOs. Informal institutions like companies hiring locals as CEOs can also curb short-termism without additional costs, saving social resources. Second, Chinese private listed companies currently seldom use equity to motivate executives. Such a short-term evaluation system has increased the short-termism of management. Therefore, it is necessary to improve the executive compensation system of Chinese private listed companies, by weighing more on the long-term incentive system. Third, government support is undoubtedly important for corporate innovation. The shortage of funds is a major restricting factor for enterprise innovation. However, the government should not only provide financial support but utilizes its public resources to provide a combined subsidy strategy to support enterprises’ long-term development. At last, regarding replication of this study in other countries, it is suspectable that the main conclusion will still hold worldwide but the exact mechanism of the CEO locality effect may differ across country samples (Bulathsinhalage and Pathirawasam, 2017; Mitan et al., 2021; Valaskova et al., 2021a). Other changes in the external conditions, such as emerging industries (Kliestik et al., 2020; Durana et al., 2021b; Lazaroiu et al., 2021; Valaskova et al., 2021c), life cycles (Durana et al., 2021a), pandemic shocks (Tijani et al., 2021), etc., may also strengthen or weaken the connection between local-province CEO identity and management myopia operations. Future studies can follow these topical directions as well as adopt novel machine learning methodologies (Krulicky and Horak, 2021; Pugliese et al., 2021) for this issue.

## Data availability statement

The original contributions presented in this study are included in the article/supplementary material, further inquiries can be directed to the corresponding author.

## Author contributions

QC and XW conceived of the presented idea, performed preliminary data collection, and took the lead in writing the first manuscript. XG and XW performed new data analysis and result interpretation. SN and QW together contributed to critical revision and final drafting the article. All authors contributed to the article and approved the submitted version.

## Appendix

**TABLE A1:** Variable definition.

Variables	Definition
Dependent variable	
*Local-province CEO*	An indicator equals one when the CEO’s native place or birthplace is in the same province as his or her company’s headquarters, and 0 otherwise
Explanatory variable	
*CutRD*	An indicator equals one if firm R&D expenditure in this year is smaller than that in last year; and 0 otherwise
*BeatByCutRD*	An indicator equals one when the following two conditions are satisfied at the same time; and 0 otherwise; (EPS − EPS_Forecast_)/(close price) is equal to or greater than 0; (EPS − EPS_Forecast_ + ΔRD)/(close price) is smaller than 0, where EPS_Forecast_ represents analysts’ median consensus forecast of EPS of the concerned company. ΔRD is the annual change of RD, where RD is defined as the ratio of R&D expenditure over total equity.
Mediating variables	
*Social capital*	The level of social capital in a Chinese province is proxied by a comprehensive rating on the province’s number and quality of societies and associations.
*GovSupport*	The level of government supports provided for stimulating corporate innovation is computed as the ratio of government funds received by a firm over its total R&D expenditure
Control variables	
*Executive ownership*	The share of ownership owned by executives and directors
*Capital change*	This change is calculated as ln(CAPX_t_) − ln(CAPX_t–1_), where CAPX denotes firm capital expenditure.
*Size*	Size is measured by ln(total assets).
*Leverage*	Leverage is measured by long-term debts over total assets.
*Tobin’s Q*	(outstanding shares × close price + non-circulating shares × BPS + long-term debts + current liabilities)/total assets
*Free cash flow*	The free cash flow for the firm (FCFF)
*Capital intensity*	Capital intensity is measured by PPE over total assets.
*Institutional ownership*	The share of ownership owned by institutional investors
*Retirement*	An indicator equals one if a CEO’s age is greater than 63 years old; and 0 otherwise
*Founder*	An indicator equals one if the founder of the company also assumes the CEO role; and 0 otherwise

**TABLE A2:** The distribution of local-province CEOs across year and province.

Year	Obs.	Local	%	Province	Obs.	Local	%
2014	307	138	44.95%	Guangdong	409	119	29.10%
2015	367	161	43.87%	Zhejiang	341	224	65.69%
2016	469	206	43.92%	Beijing	262	42	16.03%
2017	470	197	41.91%	Jiangsu	254	138	54.33%
2018	470	198	42.13%	Shandong	107	68	63.55%
Total	2,083	900	43.21%	Shanghai	100	37	37.00%
				Henan	87	39	44.83%
				Anhui	76	53	69.74%
				Sichuan	66	19	28.79%
				Fujian	53	28	52.83%
				Hubei	51	18	35.29%
				Hunan	40	21	52.50%
				Hebei	33	18	54.55%
				Jiangxi	32	24	75.00%
				Liaoning	31	19	61.29%
				Xinjiang	20	0	0.00%
				Tianjin	19	1	5.26%
				Heilongjiang	15	10	66.67%
				Gansu	13	6	46.15%
				Inner Mongolia	13	5	38.46%
				Hainan	11	0	0.00%
				Shaanxi	10	0	0.00%
				Chongqing	9	0	0.00%
				Yunnan	9	8	88.89%
				Jilin	6	1	16.67%
				Guizhou	5	2	40.00%
				Qinghai	5	0	0.00%
				Shanxi	3	0	0.00%
				Tibet	3	0	0.00%
				Total	2,083	900	43.21%

**TABLE A3:** CEO tenure and salary.

	Non-local-province CEO			Local-province CEO			Between-group difference	
Variable	Obs.	Mean	SD	Obs.	Mean	SD	*T*-test	Chi-square test
Tenure (month)	1,183	56.687	1.187	900	65.930	1.360	−5.1181***	235.278***
Salary (RMB Yuan)	1,183	981,151.4	32,939.33	900	785,292.7	29,806.12	4.0448***	29.631***

**TABLE A4:** Correlation matrix.

Variables	*BeatByCutRD*	*CutRD*	*Local-Province CEO*	*Executive ownership*	*Capital change*	*Size*	*Leverage*	*Tobin’s Q*	*Free cash flow*	*Capital intensity*	*Institutional ownership*	*Retirement*	*Founder*
*BeatByCutRD*	1.000												
*CutRD*	0.374***	1.000											
*Local-province CEO*	−0.076***	−0.082***	1.000										
*Executive ownership*	0.007	−0.022	0.011	1.000									
*Capital change*	0.060***	−0.068***	−0.045**	0.001	1.000								
*Size*	−0.043*	−0.061***	−0.018	−0.180***	0.106***	1.000							
*Leverage*	−0.038*	−0.050**	−0.036*	−0.071***	0.039*	0.433***	1.000						
*Tobin’s Q*	0.065***	0.052**	−0.052**	−0.011	−0.026	−0.373***	−0.180***	1.000					
*Free cash flow*	−0.052**	−0.058***	0.032	0.006	−0.162***	0.034	−0.059***	0.032	1.000				
*Capital intensity*	0.015	0.010	0.062***	−0.035	0.011	0.008	0.178***	−0.046**	−0.213***	1.000			
*Institutional ownership*	−0.032	−0.032	0.046**	−0.213***	−0.006	0.344***	0.122***	0.102***	0.079***	−0.028	1.000		
*Retirement*	−0.021	0.003	0.058***	−0.008	0.003	−0.004	−0.035*	−0.005	−0.028	0.078***	0.038*	1.000	
*Founder*	0.020	0.012	0.154***	0.119***	0.001	−0.236***	−0.143***	0.010	−0.049**	−0.030	−0.241***	0.092***	1.000

***, **, and * indicate significance at the 1%, 5%, and 10% level, respectively.

**TABLE A5:** Multicollinearity test.

Variables	VIF	1/VIF
Size	1.70	0.587
Institutional ownership	1.29	0.776
Leverage	1.28	0.780
Tobin’s *Q*	1.27	0.786
Founder	1.15	0.872
Capital intensity	1.10	0.907
Free cash flow	1.09	0.917
Local-province CEO	1.05	0.949
Executive ownership	1.05	0.950
Capital change	1.05	0.957
Retirement	1.02	0.977
Mean VIF	1.19	
